# Real-world experience of angiotensin receptor/neprilysin inhibitor (ARNI) usage in Thailand: a single-center, retrospective analysis

**DOI:** 10.1186/s12872-021-02145-9

**Published:** 2021-07-02

**Authors:** Wipharak Rattanavipanon, Thanyaluck Sotananusak, Fairus Yamaae, Arisa Chandrsawang, Pitchapa Kaewkan, Surakit Nathisuwan, Teerapat Yingchoncharoen

**Affiliations:** 1 Faculty of Pharmacy, Mahidol University, Bangkok, Thailand; 2 Chakri Naruebodindra Medical Institute, Faculty of Medicine Ramathibodi Hospital, Mahidol University, Samut Prakan, Thailand; 3 Division of Cardiology, Department of Internal Medicine, Faculty of Medicine Ramathibodi Hospital, Mahidol University, Bangkok, 10400 Thailand

**Keywords:** Heart failure, Sacubitril, Valsartan, Angiotensin receptor, Neprilysin inhibitor

## Abstract

**Background:**

Treatment of heart failure with reduced ejection fraction (HFrEF) has been revolutionized by angiotensin receptor/neprilysin inhibitor (ARNI). ARNI has been shown to significantly reduce morbidity and mortality in a large, randomized controlled trial. However, real-world evaluation of ARNI with a diverse population is still limited.

**Methods:**

HFrEF patients receiving angiotensin receptor/neprilysin inhibitor (ARNI) or standard HF treatment at a university hospital in Thailand were prospectively followed-up from January 2015 to December 2019. The primary outcome was a composite of all-cause mortality and heart failure hospitalization. Survival analysis and the Cox proportional hazard model were used to compare clinical outcomes between the two groups.

**Results:**

During a follow-up period of 12 months, the primary outcome occurred in 10 patients in the ARNI group (11.5%) and 28 in the standard treatment group (28.0%) (hazard ratio 0.34; 95% CI: 0.15–0.80; *p* = 0.013). After adjustment for confounding factors, ARNI was significantly associated with a significant reduction in the primary outcome (HR 0.32, 95% CI: 0.13–0.82, *p* = 0.017). In addition, ARNI was also significantly associated with a decrease in the clinical signs and symptoms of HF, including dyspnea, orthopnea, and fatigue. Orthostatic hypotension was more frequently reported among the ARNI group than among the standard treatment group. The rates of target dose achievement were comparable between the two groups.

**Conclusion:**

In real-world practice, ARNI use was associated with a significant reduction in both clinical outcomes and symptom improvement, while orthostatic hypotension was more common in patients in the ARNI group than in patients in the standard treatment group.

**Supplementary Information:**

The online version contains supplementary material available at 10.1186/s12872-021-02145-9.

## Background

Chronic heart failure (CHF) is one of the most common cardiac diseases, especially in the era of an aging society and a sedentary lifestyle. Moreover, the prevalence of HF has continuously increased in both developed and developing countries [1, 2]. HF has a high disease burden due to frequent hospital admissions, an inability to work during the decompensated stage, a high cost of care for both pharmacological and nonpharmacological treatment, and a high mortality rate. As a result, HF is currently considered a global health problem [3].

Among the various subtypes of HF, significant advances have been made in the treatment of heart failure with reduced ejection fraction (HFrEF), characterized by those with left ventricular ejection fraction (LVEF) of ≤ 40%. Overstimulation of neurohormones, particularly the renin–angiotensin–aldosterone system (RAAS) and sympathetic nervous system (SNS), has been the focus of HFrEF drug development for several decades. Through that understanding, various landmark trials have confirmed the benefits of angiotensin-converting enzyme inhibitors (ACEIs), angiotensin receptor blockers (ARBs), mineralocorticoid receptor antagonists (MRAs), and beta-blockers in reducing morbidity and mortality of HFrEF. Recently, angiotensin receptor/neprilysin inhibitor (ARNI) was found to further reduce morbidity and mortality compared to the standard treatment in a large, randomized controlled trial (RCT) and it is now recommended by various international guidelines for HFrEF management [4, 5].

Despite the significant advantages of ARNI demonstrated in an RCT, extrapolation of the efficacy and safety of this treatment into real-world practice has some limitations. First, the patient population included in that trial was mainly Caucasian patients, with only 18% Asian patients [6]. This limitation raises concern about ARNI usage in Asia in many ways. Differences in patient characteristics, such as the cause of HF, comorbidities, and body size, might influence the efficacy and safety of ARNI. Second, a run-in period was conducted in the landmark trial to assure tolerability of ARNI before randomization [6]. With a run-in period along with the strict inclusion/exclusion criteria applied in the RCT, the benefit-risk profile of ARNI in real-world situations may differ from that of the patients enrolled in the RCT. Currently, there is limited real-world evidence of ARNI in both Caucasian and Asian populations. None of these data are from the Southeast Asian region. We therefore conducted a pilot, real-world comparison of the effectiveness and safety of ARNI versus the standard treatment in a university hospital in Bangkok, Thailand.

## Methods

### Study design and setting

The study design was a retrospective cohort study conducted at Ramathibodi Hospital. The study center is a 1,500-bed, leading tertiary-care, university-affiliated, referral hospital located in the center of Bangkok, Thailand.

### Study participants

All patients who were diagnosed with HF and followed up at Ramathibodi Hospital from January 2015 to December 2019 were identified using the International Classification of Disease, Tenth Revision (ICD-10) for HF-related terms (Additional file 1: Table S1). Patients were recruited with the following inclusion criteria: age ≥ 18 years, diagnosed with HFrEF with baseline EF ≤ 40%, with regular follow-up at the study center, and sufficiently received guideline-directed medical therapy (GDMT) including beta-blockers and/or mineralocorticoid receptor antagonists. Patients were then categorized into two groups: those receiving ARNI and those receiving standard treatment. The definition of standard treatment in our study was mainly ACEIs or ARBs. Hydralazine/nitrate was also acceptable in patients for whom ACEIs/ARBs were contraindicated, especially in the setting of renal insufficiency. To assure a causal relationship between medication use and clinical outcomes in each group, we included only patients who had been receiving ARNI or standard treatment for ≥ 6 months. Patients were excluded if they met any exclusion criteria, including being transferred to other hospitals, referral patients without longitudinal follow-up information, and loss to follow-up. The study protocol was approved by the Committee for Research, Faculty of Medicine Ramathibodi Hospital Mahidol University (MURA No. 2019/1263) in 2019. Informed consent for study participation was not required based on the ethics requirements of the institution due to this being a retrospective chart review. This study complied with the principles of the Declaration of Helsinki.

### Data collection

The following data were extracted from the hospital database after the study population had been identified: key demographic data of the patients, including age, sex, comorbidities, relevant information about their HF, including New York Heart Association (NYHA) functional class, vital signs, HF medication history, intracardiac devices, and relevant laboratory tests. The LVEF value at baseline was retrieved from echocardiography reports. The latest LVEF report within 6 months was accepted if there was no echocardiography performed at baseline. Signs and symptoms of HF were also collected during the study period. Details of HF medication use, including dosage and target dose achievement, between the 2 groups were extracted.

### Outcomes of interest

The primary outcome of interest was the composite of all-cause mortality and/or hospitalization for decompensated HF within 12 months. Secondary effectiveness outcomes were changes in HF-related parameters, including HF symptoms, N-terminal pro B-type natriuretic peptide (NT-proBNP), and the 6-min walk test (6MWT). Secondary safety outcomes were adverse reactions, including hypotension, orthostatic hypotension, hyperkalemia, changes in renal function, dry cough, and angioedema. The rate of drug titration achievement between ARNI and standard treatment was also examined.

### Statistical analyses

Categorical variables of baseline characteristics and clinical outcomes were tabulated as percentages (%). Pearson’s Chi-square test was then used to identify statistical differences [7]. Continuous variables were tested for normality by the Kolmogorov–Smirnov test and then reported as the mean ± standard deviation or median and interquartile range [8]. Student’s t-test or the Mann–Whitney U test were used for continuous variables [9, 10]. Survival analysis was assessed using the Kaplan–Meier method [11]. Cox regression analysis was performed to adjust for confounding effects. An alpha value of *p* ≤ 0.05 was chosen to determine statistical significance. The 95% confidential intervals (CIs) were calculated. All statistical analyses were performed using SPSS Statistics version 21.0 (SPSS, Inc., Chicago, IL, USA).

## Results

### Study population

**Fig. 1:**
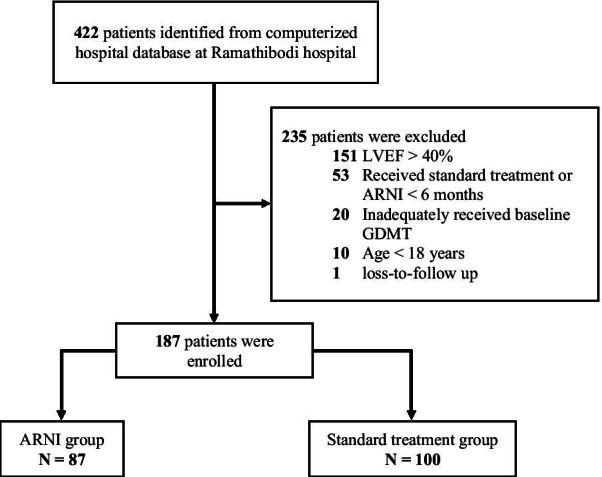
Detail of patient flow

During the study period, 422 HF patients were identified from the computerized hospital database. Two hundred thirty-five patients were excluded due to baseline EF > 40% (151 patients), being on ARNI or standard treatment for less than 6 months (53 patients), inadequate use of GDMT (20 patients), age < 18 years (10 patients), and loss to follow-up (1 patient). Therefore, 187 patients, including 87 patients in the ARNI group and 100 patients in the standard treatment group, were finally included in our pilot study. In addition, there were 10 patients receiving hydralazine/nitrate in the standard treatment group. Details of the patient flow are depicted in Fig. 1.

The baseline characteristics of the patients using ARNI and the patients using standard treatment are shown in Table 1. Overall, most baseline characteristics were similar between the groups except for the average body mass index (BMI), the prevalence of dilated cardiomyopathy (DCM), the use of cardiac resynchronization therapy (CRT) and the use of ivabradine, which were all significantly higher in the ARNI group. In contrast, chronic kidney disease (CKD) was more prevalent in the standard treatment group. Key prognostic factors of HF, including baseline NYHA functional class and baseline GDMT use, were also similar between the groups.

**Table 1:** Baseline characteristics

	ARNI n = 87	Standard treatment n = 100	*P*-value
Age (mean ± SD)	63.30 ± 15.03	59.14 ± 18.68	0.098
Male (%)	59 (67.8)	61 (61.0)	0.330
BMI (mean ± SD)	24.9 ± 6.49	22.1 ± 7.30	0.006
Baseline LVEF (mean ± SD)	26.3 ± 8.38	28.50 ± 8.07	0.078
Baseline SBP (mean ± SD)	114.3 ± 18.46	119.3 ± 19.54	0.073
Baseline DBP (mean ± SD)	69.3 ± 9.70	71.8 ± 9.91	0.085
Comorbidities (%)			
Hypertension	52 (59.8)	46 (46.0)	0.060
Diabetes	38 (43.7)	43 (43.0)	0.926
Dyslipidemia	34 (39.1)	42 (42.0)	0.685
Coronary artery disease	35 (40.2)	41 (41.0)	0.915
Atrial fibrillation	27 (31.0)	27 (27.0)	0.544
Mitral regurgitation	21 (24.1)	22 (22.0)	0.729
Chronic kidney disease	16 (18.4)	32 (32.0)	0.034
Type of cardiomyopathy (%)			
Dilated cardiomyopathy	55 (63.2)	46 (46.0)	0.018
Ischemic cardiomyopathy	27 (31.0)	41 (41.0)	0.158
NYHA classification (%)*			
Class I	12 (13.8)	22 (22.0)	0.732
Class II	56 (64.4)		
Class III	9 (10.3)	10 (10.0)	
Class IV	1 (1.2)	2 (2.0)	
Intracardiac devices (%)			
AICD	23 (26.4)	24 (24.0)	0.702
CRT	23 (26.4)	13 (13.0)	0.020
Baseline HF medication (%)			
Beta-blockers	81 (93.1)	92 (92.0)	0.775
MRA	65 (74.7)	68 (68.0)	0.312
Ivabradine	12 (13.8)	3 (3.0)	0.007
Digoxin	11 (12.6)	6 (6.0)	0.115
Diuretics	59 (67.8)	72 (72.0)	0.533

AICD = automated implantable cardioverter defibrillator, ARNI = angiotensin receptor/ neprilysin inhibitor, BMI = body mass index, CRT = cardiac resynchronization therapy, DBP = diastolic blood pressure, HF = heart failure, LVEF = left ventricular ejection fraction, MRA = mineralocorticoid receptor antagonist, NYHA = New York Heart Association, SBP = systolic blood pressure, SD = standard deviation

^*^Baseline NYHA functional class was reported in 78 and 94 patients in ARNI group and standard treatment group, respectively

### Primary outcome

**Fig. 2:**
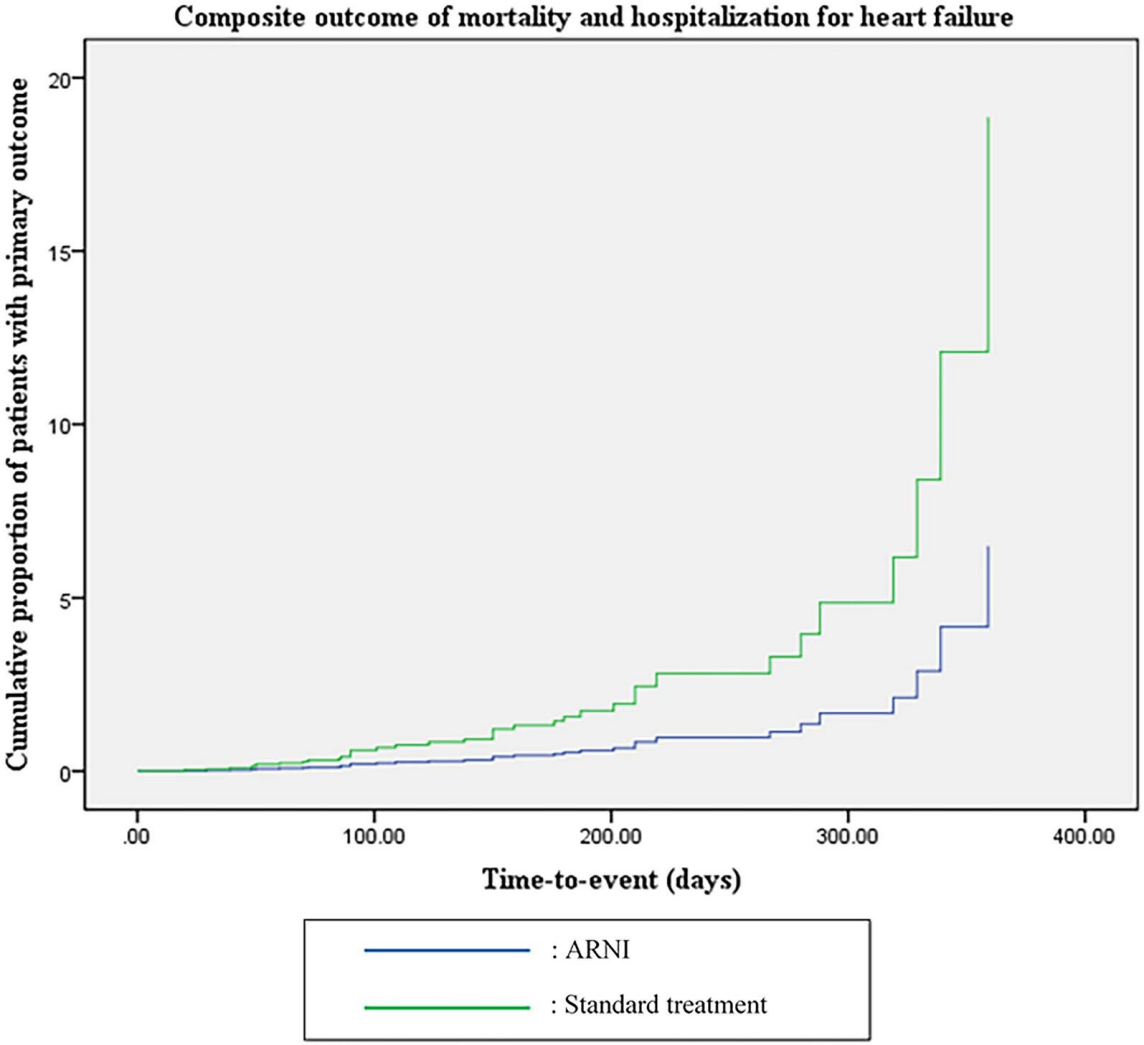
Kaplan–Meier curve of primary outcome. * Cox-regression analysis adjusted by age, BMI, dilated cardiomyopathy, chronic kidney disease, use of cardiac resynchronization therapy, use of ivabradine

During the follow-up period of 12 months, the primary outcome occurred in 10 (11.5%) and 28 (28.0%) patients in the ARNI group and standard treatment group, respectively (HR = 0.34, 95% CI = 0.15–0.80, *p* = 0.013). This difference in outcomes was driven by hospitalization for heart failure (HR = 0.31, 95% CI = 0.13–0.73, *p* = 0.007). There were 4 fatalities in the standard treatment group, while there were none in the ARNI group. After adjustment for age, BMI, dilated cardiomyopathy, chronic kidney disease, use of cardiac resynchronization therapy, and the use of ivabradine, ARNI use was associated with a significant reduction in the primary outcome, with an adjusted HR of 0.32 (95% CI = 0.13–0.82, *p* = 0.017). Details of the primary outcome and the Kaplan–Meier curves are shown in Table 2 and Fig. 2, respectively.

**Table 2:** Primary outcome

	ARNI (N = 87)	Standard treatment (N = 100)	Crude HR (95% CI), *p*-value	Adjusted HR* (95% CI), *p*-value
Primary composite outcome				
Mortality or hospitalization for heart failure	10 (11.5%)	28 (28.0%)	0.34 (0.15–0.80), *p* = 0.013	0.32 (0.13–0.82), *p* = 0.017
Component of composite outcome				
Mortality**	0 (0%)	4 (4.0%)	*p* = 0.125	–
Hospitalization for heart failure	10 (11.5%)	26 (26.0%)	0.31 (0.13–0.73), *p* = 0.007	–

ARNI = angiotensin receptor/ neprilysin inhibitor, HR = hazard ratio

^*^Cox-regression analysis adjusted by age, BMI, dilated cardiomyopathy, chronic kidney disease, use of cardiac resynchronization therapy, use of ivabradine

^**^Fisher Exact test

### Secondary outcomes

During the follow-up visits, patients in the standard treatment group had more frequent signs and symptoms of HF, including orthopnea, dyspnea, and fatigue, than those in the ARNI group. Changes in NT-proBNP and LVEF were similar between the groups (Table 3). For the secondary safety outcomes, there were no significant differences in the changes in blood pressure, sodium level, or potassium level. However, the increase in serum creatinine was significantly higher in the standard treatment group. Dry cough was more frequently reported in the standard treatment group but the difference did not reach statistical significance. No cases of angioedema were found in either group throughout the study period. Hypotension was more frequently reported in the ARNI group (13.8% vs 5.0%, *p* = 0.043), especially symptomatic orthostatic hypotension (25.3% vs 13.0%, *p* = 0.032) (Table 4). The rate of target dose achievement was similar between the two groups (55.2% in the ARNI group and 59.7% in the standard treatment group, *p* = 0.83) (Table 5).

**Table 3:** Secondary effectiveness outcomes

	ARNI (N = 87)	Standard treatment (N = 100)	*p*-value
Clinical signs and symptoms of HF (%)			
Dyspnea	6 (6.9)	22 (22.0)	0.004
Orthopnea*	5 (5.8)	16 (16.0)	0.036
Fatigue*	2 (2.3)	14 (14.0)	0.004
Edema	40 (46.0)	58 (58.0)	0.101
Changes of HF-specific parameters**			
NT-proBNP (pg/mL)	− 466 (2011)	− 81 (2341)	0.170
LVEF (%)	+ 13.0 (16.0)	+ 12.0 (16.8)	0.389

LVEF = left ventricular ejection fraction, NT-proBNP = N-terminal pro B-type natriuretic peptide,

^*^Fisher Exact test

^**^Reported as median (interquartile range)

^***^Changes of NT-proBNP was reported in 57 and 59 patients in ARNI group and standard treatment group, respectively

−Changes of LVEF was reported in 51 and 60 patients in ARNI group and standard treatment group, respectively

**Table 4:** Secondary safety outcomes

	ARNI (N = 87)	Standard treatment (N = 100)	*p*-value
Mean changes of key laboratories and vital signs			
Systolic blood pressure (mmHg)	+ 0.55 ± 16.3	+ 2.88 ± 18.1	0.355
Diastolic blood pressure (mmHg)	-0.24 ± 9.4	-0.50 ± 11.2	0.864
Serum sodium (mEq/L)	+ 0.54 ± 3.0	+ 0.20 ± 3.6	0.655
Serum potassium (mEq/L)	+ 0.17 ± 0.5	+ 0.22 ± 0.8	0.612
Serum creatinine (mg/dL)*	+ 0.04 (0.28)	+ 0.14 (0.56)	< 0.001
Clinical signs and symptoms for safety outcomes (%)			
Dry cough	7 (8.0)	11 (11.0)	0.062
Angioedema	0	0	–
Hypotension**	12 (13.8)	5 (5.0)	0.043
Symptomatic orthostatic hypotension***	22 (25.3)	13 (13.0)	0.032
Asymptomatic orthostatic hypotension***	18 (20.7)	11 (11.0)	0.068

ARNI = angiotensin receptor/ neprilysin inhibitor

^*^Reported as median (interquartile range)

^**^Fisher Exact test

^***^Orthostatic hypotension: a physical finding defined by the American Autonomic Society and the American Academy of Neurology as a systolic blood pressure decrease of at least 20 mm Hg or a diastolic blood pressure decrease of at least 10 mm Hg within three minutes of standing

**Table 5:** Characteristics of drug titration

	ARNI	Standard treatment	*p*-value
Target dose achievement	48 (55.2%)	59 (59.0%)	0.830
Average dose (mg/day)	285.7	Enalapril (23.6)	–
		Losartan (90.8)	
		Candesartan (22.7)	
		Valsartan (120)	
		Hydralazine/nitrate (95.3/31.8)	

ARNI = angiotensin receptor/ neprilysin inhibitor

### Sensitivity analysis

Sensitivity analysis excluding patients using hydralazine/nitrate in the standard treatment group was performed to evaluate the robustness of the main finding. The results of the sensitivity analysis were consistent with those of the main analysis. Details of the sensitivity analysis are shown in Additional file 2:  Table S2.

## Discussion

While data from randomized, controlled trials are the gold standard in evaluating the efficacy and safety of a treatment, real-world evaluation of the performance of such treatment can provide useful information and expand our understanding of the benefit-risk of such treatment in real clinical practice. To the best of our knowledge, this study represents the first real-world evidence evaluating ARNI in the Southeast Asia region. Our pilot study confirmed the benefit of ARNI in reducing morbidity and mortality in the HFrEF population living in this region compared to the standard treatment. This study also supports the favorable tolerability profile of ARNI in a more diverse population compared to those in the RCT.

As expected, there were both similarities and differences in the baseline characteristics of our study population compared to that of the PARADIGM-HF study. Compared to the Western population, the average BMI in our study was only 22.8–24.6 kg/m^2^ versus 28.1–28.2 kg/m^2^ in the PARADIGM-HF trial. In terms of the specific baseline prognosis of HF, the NYHA functional class was mainly class II. The baseline rates of GDMT use, including beta-blockers and MRA, were generally high and comparable between the two groups. The rate of intracardiac devices (AICD and CRT) was even higher than that of the PARADIGM-HF trial (the rates of AICD and CRT usage in the PARADIGM-HF trial were approximately 15% and 7%, respectively) [6]. This reflects an acceptable quality of HF care in Thailand.

Since the introduction of ARNI, studies assessing the benefit and risk of ARNI in real-world situation have increased [12]. For Asian population, there are currently 6 published studies. Among these studies, four studies compared pre- and post-treatment changes in surrogate markers or described safety and tolerability of ARNI initiation, without a control group [13–16]. Two studies from Taiwan compared the effectiveness and safety of ARNI versus standard treatment. Chang et al. compared clinical outcomes of 466 ARNI users to 466 patients receiving standard treatment in a single center study [17]. Another study compared 502 ARNI users to 489 patients receiving angiotensin receptor blockers based standard regimen. Patient selections was performed using propensity score matching, and the two groups were compared using inverse probability of treatment weighting (IPTW) [18]. Compared to the Taiwanese studies, patient characteristics such as age, sex, BMI, and comorbidities of this present study are relatively similar. However, the baseline rate of GDMT use and intracardiac devices in our population were higher than that of the Taiwanese studies. (beta blockers: 92% vs 80%, intracardiac devices; 25% vs 10%, respectively) [17, 18]. Despite such difference, it is reassuring to see that positive findings of our study are in concordance with Taiwanese studies along with other real-world ARNI studies in other population.

Due to the nonrandomized nature of our study, there were differences in the baseline characteristics between the two groups. Most patients in the ARNI group had dilated cardiomyopathy (DCM). The prognosis of DCM is known to be poor, especially when the LVEF is ≤ 35% and the NYHA functional class is III-IV [19]. Therefore, the prognosis of the ARNI group might be worse than that of the standard treatment group. Moreover, patients in the ARNI group had a higher rate of ivabradine and CRT use. This may indicate more symptomatic patients in the ARNI group. In contrast, more patients in the standard group had CKD, which worsens the HF prognosis [20]. Although ACEIs and ARBs have been widely used in patients with CKD, clinical evidence of ARNI use in CKD patients is still limited. This may lead to an imbalance in CKD between these 2 groups. However, we attempted to adjust for these confounding factors by using Cox regression analysis for the primary outcome.

In our pilot study, ARNI treatment was associated with a significant reduction in the composite of all-cause mortality and heart failure hospitalization compared to the standard treatment. However, this favorable outcome was primarily driven by a reduction in hospitalization. The effect size found in our study appeared to be even larger than that of the PARADIGM-HF trial [6]. Based on the Kaplan–Meier curve of the primary outcome, the effectiveness of ARNI was observed within 3 months of drug initiation. Unlike the PARADIGM-HF and other high-quality real-world evidence, our pilot study was unable to demonstrate a clear mortality benefit of ARNI due to the small sample size and short follow-up time (12 months) [6, 17, 18]. However, there was a trend toward lower mortality in patients using ARNI despite the very small number of events.

Consistent with the RCT, a significant reduction in HF signs and symptoms was also found in the ARNI group compared to the standard treatment group. While there was a trend toward a greater reduction in NT-proBNP, this did not reach statistical significance. This was partly due to significant missing data in our cohort since the measurement of NT-proBNP was sporadically implemented, which represented a variation in practice among clinicians. The changes in biochemical markers were all consistent with the known effects of ARNI. We observed that the increase in serum creatinine was significantly lower in the ARNI group. This renal outcome parallels the results from PARADIGM-HF and PARAGON-HF, which was conducted in HF patients with a preserved EF [6, 21]. ARNI has been shown to effectively preserve renal function in several studies, with possible mechanisms of antioxidant, anti-inflammatory, and antifibrotic effects through NP activation in the kidneys [22]. However, the higher incidence of CKD in the standard treatment group at baseline might partly contribute to this difference. Therefore, readers should be cautioned when interpreting this finding. As expected, hypotension was significantly higher in ARNI, especially symptomatic orthostatic hypotension. Despite the lack of a run-in period in real-world practice, angioedema was not found in any case in either arm. In terms of drug titration, patients in both groups were equally titrated to the maximum dose. These findings confirm the safety and tolerability profile of ARNI in patients with HFrEF in Thailand.

Although our pilot study revealed positive results of ARNI in real-world practice, several limitations need to be addressed. First, this was a small, real-world study with a nonrandomized design and a small number of patients. Additional large, multicenter, real-world studies should be conducted. Second, the short duration of follow-up in this study may lead to an underestimation of the mortality benefit. Therefore, future studies should have longer follow-up times to capture the mortality benefit of ARNI in real-world practice. Third, sodium-glucose cotransporter-2 (SGLT-2) inhibitors had not been approved for the treatment of HFrEF at the time of the study. Therefore, the usage of SGLT-2 inhibitors was not included in our analysis. Fourth, missing data for some secondary efficacy outcomes were significant due to the retrospective nature of the analysis. However, significant missing data occurred only among some secondary outcomes that were not strongly associated with the primary outcome. Fifth, all patients who were diagnosed with HF and followed-up at the study site during January 2015 to December 2019 were included based on the inclusion criteria. As a result, our study population contained both newly diagnosed cases and those with established disease. Finally, due to the characteristics of this observational study, our findings were susceptible to confounding factors despite our efforts to perform statistical adjustments.

## Conclusion

Our pilot study demonstrated the effectiveness and safety profile of ARNI in real-world practice. Compared to the standard treatment group, ARNI was significantly associated with a reduction in a composite of all-cause mortality and heart failure hospitalization, which was mainly driven by a reduction in hospitalization. Users of ARNI also reported fewer signs and symptoms of heart failure, without serious adverse events. Orthostatic hypotension was more frequently reported in the ARNI group. This study has confirmed the real-world effectiveness and safety of ARNI treatment in HFrEF patients in the Southeast Asia region.

## Supplementary Information

**Additional file 1: Table S1**. International classification of disease, tenth revision (ICD-10) for heart failure.

**Additional file 2: Table S2**. Sensitivity analysis excluding patients using hydralazine/nitrate.

## Data Availability

The datasets used and/or analysed during the current study are available from the corresponding author on reasonable request.

## Abbreviations

6MWT: 6-Minute walk test
ACEIs: Angiotensin-converting enzyme inhibitors
AICD: Automated implantable cardioverter defibrillator
ARBs: Angiotensin receptor blockers
ARNI: Angiotensin receptor blocker/ neprilysin inhibitor
BMI: Body mass index
CHF: Chronic heart failure
CI: Confidence interval
CKD: Chronic kidney disease
CRT: Cardiac resynchronization therapy
DCM: Dilated cardiomyopathy
EF: Ejection fraction
GDMT: Guideline-directed medical therapy
HF: Heart failure
HR: Hazard ratio
HFrEF: Heart failure with reduced ejection fraction
LVEF: Left ventricular ejection fraction
MRAs: Mineralocorticoid receptor antagonists
NT-proBNP: N-terminal pro B-type natriuretic peptide
NYHA: New York Heart Association
RAAS: Renin–angiotensin–aldosterone system
RCTs: Randomized controlled trials
SGLT-2: Sodium-glucose cotransporter-2
SNS: Sympathetic nervous system
