# Supplementary material for: Real-world experience of angiotensin receptor/neprilysin inhibitor (ARNI) usage in Thailand: a single-center, retrospective analysis
Source: BMC Cardiovasc Disord. 2021 Jul 2;21:324. doi: 10.1186/s12872-021-02145-9 (PMC8254265; doi:10.1186/s12872-021-02145-9)
Supplement: Supplementary file 1 — Additional file 1: Table S1. International classification of disease, tenth revision (ICD-10) for heart failure. [file 12872_2021_2145_MOESM1_ESM.docx]

**Table S1 International Classification of Disease, Tenth Revision (ICD-10) for heart failure**

| Code | Condition |
| --- | --- |
| I50.1 | Left ventricular failure, unspecified |
| I50.2 | Systolic congestive heart failure |
| I50.4 | Combined systolic and diastolic heart failure |
| I50.82 | Biventricular heart failure |
| I50.83 | End-stage heart failure |
| I11.0 | Hypertensive heart disease with heart failure |
| I13.0 | Hypertensive heart and chronic kidney disease with heart failure |
| I42 | Cardiomyopathy |
| I42.0 | Dilated cardiomyopathy |
| I42.4 | Endocardial fibroenlastosis |
| I42.5 | Restrictive cardiomyopathy |
| I42.6 | Alcohol cardiomyopathy |
| I42.7 | Cardiomyopathy due to drug and external agent |
| I42.8 | Other cardiomyopathy |
| O90.3 | Peripartum cardiomyopathy |
