# Supplementary material for: Real-world experience of angiotensin receptor/neprilysin inhibitor (ARNI) usage in Thailand: a single-center, retrospective analysis
Source: BMC Cardiovasc Disord. 2021 Jul 2;21:324. doi: 10.1186/s12872-021-02145-9 (PMC8254265; doi:10.1186/s12872-021-02145-9)
Supplement: Supplementary file 2 — Additional file 2: Table S2. Sensitivity analysis excluding patients using hydralazine/nitrate. [file 12872_2021_2145_MOESM2_ESM.docx]

**Table S2 Sensitivity analysis excluding patients using hydralazine/nitrate**

|  | **ARNI**  **(N = 87)** | **Standard treatment**  **(N = 90)** | **Crude HR**  **(95% CI),**  **p-value** | **Adjusted HR* (95% CI),**  **p-value** |
| --- | --- | --- | --- | --- |
| **Primary composite outcome** | | | | |
| Mortality or hospitalization for heart failure | 10 (11.5%) | 25  (27.8%) | 0.35  (0.15-0.83),  p = 0.017 | 0.33  (0.13-0.83),  p = 0.019 |
| **Component of composite outcome** | | | | |
| Mortality** | 0 (0%) | 3 (3.3%) | p = 0.246 | - |
| Hospitalization for heart failure | 10 (11.5%) | 25 (27.8%) | 0.29  (0.12-0.68),  p = 0.005 | - |

ARNI = angiotensin receptor/ neprilysin inhibitor, HR = hazard ratio

* Cox-regression analysis adjusted by age, BMI, dilated cardiomyopathy, chronic kidney disease, use of cardiac resynchronization therapy, use of ivabradine

** Fisher Exact test
